# Thermodynamics of Fatigue: Degradation-Entropy Generation Methodology for System and Process Characterization and Failure Analysis

**DOI:** 10.3390/e21070685

**Published:** 2019-07-12

**Authors:** Jude A. Osara, Michael D. Bryant

**Affiliations:** Mechanical Engineering Department, The University of Texas at Austin, Austin, TX 78712, USA

**Keywords:** fatigue, system failure, degradation analysis, entropy generation, stress strain, plastic strain, thermodynamics, health monitoring

## Abstract

Formulated is a new instantaneous fatigue model and predictor based on ab initio irreversible thermodynamics. The method combines the first and second laws of thermodynamics with the Helmholtz free energy, then applies the result to the degradation-entropy generation theorem to relate a desired fatigue measure—stress, strain, cycles or time to failure—to the loads, materials and environmental conditions (including temperature and heat) via the irreversible entropies generated by the dissipative processes that degrade the fatigued material. The formulations are then verified with fatigue data from the literature, for a steel shaft under bending and torsion. A near 100% agreement between the fatigue model and measurements is achieved. The model also introduces new material and design parameters to characterize fatigue.

## 1. Introduction

All solids can yield or fail under continuous loading. For static loading, equilibrium and monotonic conditions facilitate evaluation of a component’s strength. For dynamic loading, assessment of degradation leading to fatigue failure is complicated by various dynamic loads, material composition and load conditions. With metals under heavy structural loading, sudden failure can be catastrophic [1]. Cyclic loading causes about 90% of all metal failures [2,3,4,5,6,7]. Thermal cycle-induced stresses can fatigue electronic components.

Common fatigue analysis methods include stress-life (Wohler) curves for high-cycle fatigue (HCF) and strain-life curves for low-cycle fatigue (LCF). Vasudevan et al. [8] discussed deficiencies in structural fatigue life models involving crack growth *da/dN* and the challenges in implementing these models. Existing approaches sometimes give inconsistent results, and failure measures are usually component- or process-specific. Recent entropy-based fatigue studies [9,10,11,12,13,14,15,16,17,18,19,20,21,22,23] have shown high accuracy, establishing thermodynamic energies and entropies as measures of system damage, degradation and failure [7,24].

### Thermodynamics-Based Fatigue Models

Lemaitre and Chaboche [7] coupled damage mechanics with irreversible thermodynamics to present a comprehensive breakdown of elastic, elastoplastic and elastoviscoplastic behavior of solids, and considered spatial rate-dependent and rate-independent response to loading. Chaboche [25,26] presented constitutive relations for isotropic and kinematic hardening (or softening) of metals, with experimental data obtained for stainless steel. Investigating size effects in low-cycle fatigue of solder joints, Gomez and Basaran [9,10] formulated thermodynamic models for isotropic and kinematic hardening, verified with experiments and finite elements. Via simulations and measurements, Basaran et al. [11,12,13] directly related entropy to damage evolution in solids. Combining Boltzmann’s entropy S=klnW as a measure of molecular disorder with Prigogine’s entropy balance $dS=dS_{e}+dS\prime$, the authors defined a continuum damage mechanics damage variable

(1) $$D=D_{cr}\frac{W-W_{0}}{W}=D_{cr}\left[1-e^{-\left(m/R\right)\left(s-s_{0}\right)}\right]$$

similar to Einstein’s oscillator energy of a nonmetallic crystalline solid [27]. Equation (1), where *D_cr_* = critical disorder coefficient, *W* = disorder parameter, *m* = specific mass and *R* = gas constant, gives damage as a function of specific entropy change

(2) $$s-s_{0}=\int_{t_{0}}^{t}\frac{\sigma \;:\varepsilon_{p}}{T\rho }dt+\int_{t}^{t_{0}}\frac{k}{\rho }\frac{{\left|grad\;T\right|}^{2}}{T^{2}}dt+\int_{t_{0}}^{t}\frac{r}{T}dt.$$

Khonsari, Amiri and Naderi [14,23] related entropy to mechanical fatigue via extensive experiments and data, and proposed fatigue fracture entropy *FFE* as a consistent material property independent of load type, cycle frequency, amplitude or specimen size. Using thermodynamic formulations by Lemaitre and Chaboche [7], Khonsari et al. presented entropy generation rate

(3) $$\dot{S^{\prime }}=\frac{{\dot{W}}_{p}}{T}-\frac{A_{k}{\dot{V}}_{k}}{T}-J_{q}\frac{grad\;T}{T^{2}}\geq 0$$

where the first right-hand side term is the plastic strain entropy from plastic strain energy *W_p_*, the second term is the non-recoverable energy and the third term is heat conduction entropy. Assuming negligible non-recoverable energy and neglecting heat conduction within the specimen, the second and third right side terms were set to zero to give $\dot{S^{\prime }}=\frac{{\dot{W}}_{p}}{T}$. By integrating up to the time of failure *t_f_*, *FFE* was obtained as

(4) $${S^{\prime }}_{TF}=\int_{0}^{t_{f}}\frac{W_{p}}{T}dt.$$

Data from bending and torsional fatigue measurements and Finite Element Analysis validated the constant process-independent, material-dependent *FFE*. Similar to Doelling et al. [28] for wear, the authors showed a linear interdependence between normalized entropy generation and normalized number of cycles as

(5) $$\frac{s_{i}}{s_{g}}\approx \frac{N}{N_{f}}$$

where $s_{i}$ and $s_{g}$ are entropies at cycles *N* and failure $N_{f}$, respectively. Results came from over 300 specimens. Through Equation (5), damage accumulation parameter *D* [29] was related to entropy generation. Naderi and Khonsari [16] applied the approach in reference [15] to variable loading and proposed a universally consistent damage accumulation model. Amiri et al. [18] replaced entropy generation from plastic energy dissipation with entropy transfer out of the loaded specimen via heat. With thermal energy balance, heat transfer out of the specimen into the surroundings was evaluated from measurements of specimen and ambient temperatures during loading via

(6) $$\left(\oint^{\text{​}}\sigma_{ij}d\varepsilon_{ij}\right)f={\dot{H}}_{cd}+{\dot{H}}_{cv}+{\dot{H}}_{rd}+\rho c_{p}\frac{\partial T}{\partial t}+{\dot{E}}_{p}$$

where the first three right side terms represent heat transfer via conduction, convection and radiation. The authors described the last two right side terms as variation of internal energy, comprised of temperature-dependent change and a “cold” microstructural change assumed negligible at steady state, to simplify evaluation of entropy flow rate. They reported an uncertainty of 7.8% in their entropy values. Naderi and Khonsari [17] later developed a real-time fatigue monitoring system. With *FFE*$\left(\gamma_{f}\right)$ as failure parameter and a failure criterion $\gamma \leq 0.9\gamma_{f}$, failure was consistently predicted with about 10% error, attributed to the difference between temperature measurement location on the sample and actual failure location. Naderi and Khonsari [19] demonstrated entropy-based fatigue analysis methods more consistent under varying load conditions than stress- and hysteresis energy-based models. Naderi and Khonsari’s [20,21] entropy-based fatigue failure indicated stored energy in composite laminates comparable to dissipated heat, leading to the inclusion in their formulations of heat storage entropy and a crack-initiating damage entropy. Using hysteresis energy balance, entropy accumulation was

(7) $$S\prime =\int_{0}^{tf}\frac{E_{th}}{T}+\int_{0}^{tf}\frac{E_{diss}}{T}+\int_{0}^{tf}\frac{E_{d}}{T}$$

where $E_{th}$ is heat stored, $E_{diss}$ is heat dissipated, and $E_{d}$ is damage energy. Combining the first two terms of Equation (7) as mechanical entropy, experimental results compared each entropy component to the total entropy.

Russian works selected by Sosnovskiy and Sherbakov in reference [30] described the inadequacies of existing models in characterizing complex damage of tribo-fatigue systems due to simultaneously occurring degradation mechanisms, e.g., sliding friction, fretting, impact, corrosion, heating, etc. Using a cumulative general damage term $\omega^{\prime }$ $\left(0<\omega^{\prime }<1\right)$ including mechanical, thermal and electrochemical energy changes, they proposed a tribo-fatigue entropy

(8) $${S^{\prime }}_{TF}=\omega \prime \frac{dW_{D}}{T}$$

where $W_{D}$ is the absorbed damage energy at the failure site. Total entropy change summed thermodynamic entropy change and tribo-fatigue entropy, Equation (8), as

(9) $$dS_{T}=dS+dS_{TF}=\frac{dU}{T}+\frac{\delta W}{T}-\frac{\mu dN^{\prime }}{T}+\omega \prime \frac{dW_{D}}{T}$$

where the first right side term is internal energy change, the second term is boundary work, the third is chemical reaction and the fourth is damage. The authors related $\omega^{\prime }$ to normalized time and predicted human death via stress/damage accumulation from birth, depicting an exponential relationship. They presented a human life version of the Wohler (S-N) curve showing a profile similar to metals. Naderi et al.’s Equation (7) and Sosnovskiy et al.’s Equation (9) are equivalent formulations of entropy evolution (with *dN’* = 0 in Equation (9)). Direct comparison shows damage energy $dE_{D}=\omega \prime dW_{D}$. Sosnovskiy et al. [31] further expanded and combined the above formulations with continuum damage mechanics to form mechanothermodynamics (MTD). Their data for isothermal fatigue of steel indicated an error of 15%.

Extensive data showed consistency of entropy measurements in estimating mechanical damage and failure in dynamically loaded components. Currently, most fatigue-entropy formulations apply to metal and composite laminate fatigue under mechanical loading only. Via thermodynamic principles and the DEG theorem, this article relates existing fatigue damage measures to instantaneous active process entropies to derive a fatigue model consistent with thermodynamics and natural laws. Data [15,18,32] will verify this DEG approach.

Subsequent sections are as follows:

- Section 2 introduces and reviews the DEG theorem and procedure.
- Section 3 reviews thermodynamics and introduces phenomenological entropy, consisting of a boundary work component and an internal fluctuation component.
- Section 4 couples fatigue analysis to thermodynamics.
- Section 5 uses published experimental data to validate and visualize the model.
- Section 6 discusses results and the models.
- Section 7 summarizes and concludes.

## 2. Degradation-Entropy Generation Theorem Review

In accordance with Rayleigh’s dissipation function of mechanics [33], Onsager’s reciprocity theorem in irreversible thermodynamics [34] and Prigogine’s dissipative structures [35,36], a quantitative study of degradation of systems by dissipative processes [24] formulated the Degradation-Entropy Generation DEG theorem, establishing a direct relation between material/system degradation and the irreversible entropies produced by the dissipative processes that drive the degradation. Entropy measures disorganization in materials. Since degradation is advanced and permanent disorganization, entropy generation is fundamental to degradation.

### 2.1. Statement

Given an irreversible material transformation caused by *i =* 1,2,…, *n* underlying dissipative processes and characterized by an energy, work, or heat $p_{i}$. Assume effects of the mechanism can be described by an appropriately chosen variable

(10) $$w=w\left(\;p_{i}\;\right)=w\left(\;p_{1},\;p_{2},\;\ldots \;,\;p_{n}\right),\;i=1,2,\ldots ,n$$

that measures the material transformation and is monotonic in the effects of each $p_{i}$. Then the rate of degradation

(11) $$\dot{w}=\underset{i}{\sum }B_{i}\dot{S}\prime_{i}$$

is a linear combination of the rates of the irreversible entropies $\dot{S}\prime_{i}$ generated by the dissipative processes $p_{i}$, where the degradation/transformation process coefficients

(12) $$B_{i}={\frac{\partial w}{\partial S\prime_{i}}|}_{p_{i}}$$

are slopes of degradation *w* with respect to the irreversible entropy generations $S\prime_{i}$ $=S\prime_{i}\left(\;p_{i}\right),$ and the ${|}_{p_{i}}$ notation refers to the process $p_{i}$ being active. The theorem’s proof [24] is founded on the second law of thermodynamics. Integrating Equation (11) over time yields the total accumulated degradation

(13) $$w=\underset{i}{\sum }B_{i}S\prime_{i}$$

which is also a linear combination of the accumulated entropies $S\prime_{i}$.

### 2.2. Generalized Degradation Analysis Procedure

Bryant et al.’s [24] structured DEG theorem-based degradation analysis methodology embeds the physics of the dissipative processes into the energies $p_{i}=p_{i}\left(\zeta_{ij}\right),\;j=1,2,\ldots ,m$. Here the *p_i_* can be energy dissipated, work lost, heat transferred, change in thermodynamic energy (internal energy, enthalpy, Helmholtz or Gibbs free energy) or some other functional form of energy, and the $\zeta_{ij}$ are time-dependent phenomenological variables (loads, kinematic variables, material variables, etc.) associated with the dissipative processes *p_i_*. The approach

(1) identifies the degradation measure *w*, dissipative process energies $p_{i}$ and phenomenological variables $\zeta_{ij}$,
(2) finds entropy generation S′ caused by the $p_{i}$,
(3) evaluates coefficients $B_{i}$ by measuring increments/accumulation or rates of degradation versus increments/accumulation or rates of entropy generation, with process $p_{i}$ active.

This approach can solve problems consisting of one or many variegated dissipative processes. Previous applications of the DEG theorem analyzed friction and wear [24,37,38] and metal fatigue [15,18,22,39] grease degradation [32] and battery aging [40].

## 3. Thermodynamic Formulations

This section reviews the first and second laws of thermodynamics applied to real systems [27,36,41,42,43,44,45,46].

### 3.1. First Law—Energy Conservation

The first law

(14) $$dU=\delta Q-\delta W+\sum \mu_{k}dN_{k}$$

for a stationary thermodynamic system neglecting gravity, balances *dU* the change in internal energy, *δQ* the heat exchange across the system boundary, *δW* the energy transfer across the system boundary by work, and ${\sum^{\text{​}}\mu }_{k}dN_{k}$ the internal energy changes due to chemical reactions, mass transport and diffusion, where $\mu_{k}$ are chemical, flow and diffusion potentials, $N_{k}=N\prime_{k}+N^{e}{}_{k}+N^{d}{}_{k}$ are numbers of moles of species *k* with $N\prime_{k}$, $N^{e}{}_{k}$ and $N^{d}{}_{k}$ the reactive/diffusive and transferred species respectively. Inexact differential δ indicates path-dependent variables. For chemical reactions governed by stoichiometric equations, ${\sum^{\text{​}}\mu }_{k}dN_{k}=Ad\xi$ [36,43,47] where *A* is reaction affinity and dξ is reaction extent.

### 3.2. Second Law and Entropy Balance—Irreversible Entropy Generation

Known as the Clausius inequality, the second law of thermodynamics states: The change in closed system entropy

(15) $$dS\geq \frac{\delta Q}{T},$$

equal to or greater than the measured entropy transfer across the system boundary via heat. For open systems (having mass flow), the right side of Equation (15) would include a mass transfer term. For a reversible process

(16) $$dS=dS_{rev}=\frac{\delta Q_{rev}}{T}$$

approximates a quasi-static (very slow) process in which total entropy change occurs via reversible heat transfer $\delta Q_{rev}$. The second law as the equality $dS=\delta S_{e}+\delta S^{\prime }$ [12,34] equates the change in entropy *dS* to the measured entropy flow $\delta S_{e}$ across the system boundaries from heat transfer and/or mass transfer (for open systems), plus any entropy $\delta S^{\prime }$ produced within the system boundaries by dissipative processes. Entropy generation $\delta S^{\prime }$ measures the permanent changes in the system when the process constraint is removed or reversed [27,43], allowing the system to evolve. For a closed system [11,33]

(17) $$dS=dS_{irr}=\frac{\delta Q}{T}+\delta S^{\prime }$$

where $dS_{irr}$ is entropy change via an irreversible (real) path, *δQ*/*T* is entropy flow by heat transfer which may be positive or negative, and *T* is the temperature of the boundary where the energy/entropy transfer takes place. The second law also asserts entropy generation $\delta S^{\prime }\geq 0$.

### 3.3. Combining First and Second Laws with Helmholtz Potential

For a system undergoing quasi-static heat transfer and compression work, Equation (14) with δQ = $\delta Q_{rev}=TdS_{rev}$ from Equation (16) becomes [45]

(18) $$dU=TdS_{rev}-P_{rev}dV+{\sum^{\text{​}}\mu }_{k,rev}dN_{k}\;.$$

Here *P* is pressure and V is volume. Replacing entropy *S* with temperature *T* as the independent variable via a Legendre transform results in the Helmholtz free energy

(19) A=U−TS,

an alternate form of the first law which can measure maximum work obtainable from a thermodynamic system. Differentiating Equation (19) and substituting Equation (18) for *dU* into the result give the Helmholtz fundamental relation

(20) $$dA=dA_{rev}=-S_{rev}dT-P_{rev}dV+{\sum^{\text{​}}\mu }_{k,rev}dN_{k}\;,$$

the quasi-static change in Helmholtz energy between two states, valid for all systems. Here $dA=dA_{rev}$ is the free energy change via the reversible (*rev*) path, maximum for energy transfer out of the system and minimum for energy transfer into the system.

Via the thermodynamic State Principle, the change in system energy/entropy due to boundary interactions and/or compositional transformation is path-independent. The change can be determined via reversible (linear) or irreversible (nonlinear) paths between system states. Equality of Equations (16) and (17) is based on this principle. Eliminating *δQ* from Equation (14) via Equation (17) gives, for compression work PdV, [36,37,38,42,43]

(21) $$dU=dU_{irr}=TdS_{e}-T\delta S^{\prime }-PdV+{\sum^{\text{​}}\mu }_{k}dN_{k},$$

where reversible entropy change $dS_{rev}$ was replaced by entropy flow $dS_{e}$ and entropy generation $\delta S^{\prime }$. Differentiating Equation (19) and substituting Equation (21) for *dU* into the result give the irreversible form of the Helmholtz fundamental relation

(22) $$dA=dA_{irr}=-SdT-PdV+{\sum^{\text{​}}\mu }_{k}dN_{k}-T\delta S^{\prime }\leq 0$$

where $dA=dA_{irr}$ is the free energy change via irreversible (*irr*) path, maximum for energy transfer out of the system and minimum for energy transfer into the system. Equations (20) and (22) are equivalent representations of total change in Helmholtz free energy of all active systems, and show *dA* can be evaluated via an idealized change $dA_{rev}$, or a real spontaneous evolution $dA_{irr}$. From Equation (22), define phenomenological Helmholtz free energy change

(23) $$dA_{phen}=-SdT-PdV+{\sum^{\text{​}}\mu }_{k}dN_{k},$$

due only to changes in measurable intensive and extensive properties of a real system. With a known $dA_{rev}$, Equations (20) and (22) are combined to give

(24) $$\delta S^{\prime }=-\frac{SdT}{T}-\frac{PdV}{T}+\frac{{\sum^{\text{​}}\mu }_{k}dN_{k}}{T}-\frac{dA_{rev}}{T}\geq 0$$

which satisfies the second law. During energy extraction or loading, $dT\geq 0,dV\geq 0,dN_{k}\leq 0$ and $dA_{rev}\leq 0$, rendering $\delta S^{\prime }\geq 0$. During energy addition or product forming process, $dT\leq 0,dV\leq 0,dN_{k}\geq 0$ and $dA_{rev}\geq 0$, reversing the signs of the middle terms in Equation (24) to preserve $\delta S^{\prime }\geq 0$ [43].

Equation (24) defines entropy generation or production as the difference between phenomenological $\delta S_{phen}=\frac{dA_{phen}}{T}=-\frac{SdT}{T}-\frac{PdV}{T}+\frac{{\sum^{\text{​}}\mu }_{k}dN_{k}}{T}$ and reversible $dS_{rev}=\frac{dA_{rev}}{T}$ entropies

(25) $$\delta S^{\prime }=\delta S_{phen}-dS_{rev}\geq 0$$

where for energy extraction $dS_{rev}\leq \delta S_{phen}<0$, and for energy addition $0<dS_{rev}\leq \delta S_{phen}.$

Comparing Equations (16) and (17), (20) and (22), verifies that changes in entropy and energy between two states are path-independent, i.e.,

(26) $$dS=dS_{rev}=dS_{irr}=\delta S_{phen}-\delta S^{\prime };\;dA=dA_{rev}=dA_{irr}=dA_{phen}-T\delta S^{\prime }.$$

In Equation (26), the change in Helmholtz energy $dA=dA_{rev}$ and entropy $dS=dS_{rev},$ evaluated for a reversible path requires only beginning and end state measurements of system variables. Contrast this for an irreversible path, wherein $dA=dA_{irr}=\delta A_{phen}-T\delta S^{\prime }$ and $dS=dS_{irr}=\delta S_{phen}-\delta S^{\prime }$ require instantaneous account of all active processes. Now dA and *dS* can be negative or positive, depending on energy flow *TdS_e_* or entropy flow *dS_e_* across system boundaries. Since neither *dA* nor *dS* measures the permanent changes in the system, this limits success of energy and entropy formulations in characterizing measurable permanent system changes. On the other hand, entropy generation, Equation (24) or (25), evolves monotonically per the second law. With $\delta S^{\prime }=0$ indicating an idealized system-process interaction, Equation (25) also indicates that a portion of any real system’s energy is always unavailable for external work, $\delta S^{\prime }>0$. Equation (25) which gives the entropy generated by the system’s internal irreversibilities alone, is in accordance with experience, similar to the Gouy-Stodola theorem of availability (exergy) analysis [44,46,48,49]. The foregoing equations are in accord with the IUPAC convention of positive energy into a system.

### 3.4. Entropy Content S and Internal Free Energy Dissipation “−SdT“

The Helmholtz fundamental relation, Equations (20) and (22), introduced “−SdT”, free energy dissipated and accumulated internally by a loaded component, which can include effects of plastic work, chemical reaction heat generation and heat from an external source. Temperature change *dT* is driven by the system entropy content *S.* Equation (20) suggests Helmholtz-based entropy of a compressible system S=S(T,V,N) depends on temperature *T,* volume V and number of moles N. Via partial derivatives

(27) $$dS={\left(\frac{\partial S}{\partial T}\right)}_{V,N}dT+{\left(\frac{\partial S}{\partial V}\right)}_{T,N}dV+{\left(\frac{\partial S}{\partial N}\right)}_{T,V}dN.$$

From Maxwell’s thermodynamic manipulation of mixed partial second derivatives and Callen’s derivatives reduction technique [27], Equation (27) can be re-stated using established and measurable system parameters [27,36]

(28) $${\left(\frac{\partial S}{\partial T}\right)}_{V,N}=\frac{C_{V}}{T};\;{\left(\frac{\partial S}{\partial V}\right)}_{T,N}={\left(\frac{\partial P}{\partial T}\right)}_{V,N}=\frac{\alpha }{\kappa_{T}};\;{\left(\frac{\partial S}{\partial N}\right)}_{T,V}=-{\left(\frac{\partial \mu }{\partial T}\right)}_{V,N}$$

where $C_{V}$ is heat capacity (for solids, $\;C_{P}\approx C_{V}=C$), $\alpha =\frac{1}{V}\;{\left(\frac{\partial V}{\partial T}\right)}_{P,N}$ is the volumetric coefficient of thermal expansion and $\kappa_{T}=-\frac{1}{V}\;{\left(\frac{\partial V}{\partial P}\right)}_{T,N}$ is isothermal compressibility. For a constant-composition system (no independent chemical transformations or phase changes), ${\left(\frac{\partial \mu }{\partial T}\right)}_{V,N}=0$, to give

(29) $$dS=\frac{C}{T}dT+\frac{\alpha }{\kappa_{T}}dV.$$

Integrating with initial condition $S_{0}=0$ gives entropy content

(30) $$S=C\ln T+\frac{\alpha }{\kappa_{T}}V$$

and internal free energy dissipation

(31) $$-SdT=-\left(C\ln T+\frac{\alpha }{\kappa_{T}}V\right)dT.$$

## 4. Differential/Elemental Fatigue Analysis

The foregoing formulations will be applied to a component under cyclic mechanical, thermal and chemical loading [40].

### 4.1. Local Equilibrium

An extensively verified theorem by Prigogine [35,43,50] hypothesized that every macroscopic system is made up of elemental volumes wherein observable system properties can be instantaneously ascertained, and established equilibrium formulations valid for each elemental volume. If continuity or thermodynamic contact exists between measurement location and the region of interest, the evolution of locally defined state variables can adequately characterize the overall transformation of the component.

### 4.2. Helmholtz Energy Dissipation and Entropy Generation

Engineering Model: Thermodynamic boundary encompasses system only; loading occurs across system boundary; system is closed; heat transfers with surroundings (system is not isolated). Equation (22) gives the loss of Helmholtz energy in a compressible system. To represent all forms of dynamic loading, thermodynamic boundary work δW = *YdX* replaces compression work δW=PdV. Here *Y* is generalized constraint/force/load potential, *X* is generalized response/displacement/loading, ${\sum^{\text{​}}\mu }_{k}dN_{k}$ (= μdN for a closed system with one reactive component) defines energy loss due to independent chemical processes such as corrosion or radioactive decay, where $dN=\frac{dm}{M_{m}}$, m is the component’s mass and *M_m_* is molecular mass. Equation (24) with generalized boundary loading and active chemical reaction

(32) $$\delta S^{\prime }=-\frac{SdT}{T}-\frac{YdX}{T}+\frac{\mu dm}{M_{m}T}-\frac{dA_{rev}}{T}\geq 0$$

accumulates entropy generation of three simultaneous active processes. Note that derivations involving pressure-volume work in Equation (18) and subsequent Equations such as (27) and (29) originated from the general work term *δW* in the first law, Equation (14). Reformulating with generalized force-displacement work *YdX* instead of pressure-volume work PdV allows replacement of pressure and volume terms in these formulations, without loss of generality.

Using generalized directional boundary work *YX*, Equation (30) gives entropy content

(33) $$S=C\ln T+\frac{\alpha }{\kappa_{T}}X$$

which evolves monotonically in all systems. Note that the assumption of zero initial entropy content $S_{0}$ in Equation (33) is considered valid in a new component without defect, for analytical and characterization purposes. The first right side term is entropy from temperature changes (thermal energy storage). The second term emanates from internal changes in structure and configuration. Here generalized system/material properties $C=T{\left(\frac{\partial S}{\partial T}\right)}_{Y}>0$, $\alpha =\frac{1}{X}{\left(\frac{\partial X}{\partial T}\right)}_{Y}$ and $\kappa_{T}=-\frac{1}{X}{\left(\frac{\partial X}{\partial Y}\right)}_{T}>0$ are obtained as in Equation (28). While *C* and α measure system response to heat and temperature changes, generalized $\kappa_{T}$ represents isothermal *loadability*, a measure of the material/component’s “cold” response to boundary loading, which for a compressible system is compressibility.

### 4.3. Stress and Strain as Thermodynamic Variables

Most fatigue damage analyses involve evaluation of the impact of loading on a component. Energy-based formulations often define boundary work (e.g., thermal or mechanical cycling) as a volume integral of stress tensor σ times strain tensor ε with elastic and plastic components $\sigma =\sigma_{e}+\sigma_{p}$ and $\varepsilon =\varepsilon_{e}+\varepsilon_{p}$. For a non-reactive system undergoing boundary work σ:dε [7], Equation (31) becomes

(34) $$-SdT=-\left(C\ln T+\frac{\alpha }{\kappa_{T}}V\varepsilon \right)dT.$$

To clearly indicate the combined effect of thermal and structural changes due to loading, internal energy dissipation −*SdT*, expressed in terms of measured variables T, σ, ε in Equation (34), is named MicroStructuroThermal (MST) energy dissipation [32]. Here $\kappa_{T}=\frac{\partial \varepsilon_{e}}{\partial \sigma }$ is the isothermal *strainability* where $\varepsilon_{e}$ is elastic strain and σ is stress. Similar to application in compression work, $\kappa_{T}$ can be evaluated via the inverse of elastic or torsional modulus for normal or torsional loading. Torsional and frictional loads are described using shear stress τ and shear strain γ tensors. Similar terms as in Equation (34) were derived by Morris [51].

#### 4.3.1. Cyclic Loading—High-and Low-Cycle Fatigue

Elastoplastic strain response to tensile stress is often modeled via the Ramberg-Osgood relation [52]: $\varepsilon =\frac{\sigma }{E}+K{\left(\frac{\sigma }{E}\right)}^{n}$. Fatigue failure results from dynamic loading. Fatigue measurements determine strain response to stress-controlled loading or stress response to strain-controlled loading. For stress-or strain-controlled cyclic loading, Morrow [53] experimentally showed that the corresponding strain or stress amplitude and strain energy are nearly constant throughout, except for the first few cycles, and last cycles before failure [7]. In systems subject to fatigue failure (high- and low-cycle fatigue HCF and LCF), the plastic component of the response to loading is significant (predominant in LCF), especially at critical locations on the system. To account for elastic and plastic loads, cyclic strain amplitude as a function of applied stress amplitude is [53] $\varepsilon_{a}=\frac{\sigma_{a}}{E}+\varepsilon \prime_{f}{\left(\frac{\sigma_{a}}{\sigma \prime_{f}}\right)}^{1/n\prime }$ where the first right side term is elastic strain and the second is plastic strain. Via the Coffin-Manson relation, this can be restated as [54,55,56]

(35) $$\varepsilon_{a}=\frac{\sigma \prime_{f}}{E}{\left(2N_{f}\right)}^{b}+\varepsilon \prime_{f}{\left(2N_{f}\right)}^{c}$$

where $N_{f}$ is the number of cycles to failure and $2N_{f}$ is the number of strain reversals. Here *b* and *c* are fatigue strength and ductility exponents. Cyclic elastic strain energy density $W_{e}=\sigma_{N}:\varepsilon_{eN}$ is often negligible in very low cycle failure [14,15,16,17,18,19,20,21,22,23,53]. Cyclic plastic strain energy density was given by Morrow [53] as

(36) $$W_{p}=\sigma_{N}:\varepsilon_{pN}\left(\frac{1-n\prime }{1+n\prime }\right)$$

where *n’* is the cyclic strain hardening coefficient. With units J/m^3^ equivalent to Pa, energy density is often described in mechanics as toughness [53]. Combining with cyclic elastic work gives the total cyclic boundary work or strain energy density

(37) $$W=W_{e}+W_{p}=\sigma_{N}:\left[\varepsilon_{eN}+\varepsilon_{pN}\left(\frac{1-n\prime }{1+n\prime }\right)\right].$$

For cyclic loading conditions, differential cyclic time or period [57]

(38) $$dt_{N}=\frac{dt}{N_{dt}}=\frac{1}{h}$$

where *h* is the load cycle frequency and $N_{dt}$ is the number of cycles in time increment *dt*. Fatigue loads are often defined per cycle as sinusoids with stress/strain amplitude or range per cycle. Here *dt* is replaced by $N_{dt}dt_{N}$ in integrals, such as upcoming Equation (47), for convenience and compatibility with differential thermodynamic formulations such as Equation (32), as done by Meneghetti [57] and Morris [51]. The measurement time step *dt* is often greater than *dt_N_* when measuring phenomenological variables or parameters such as temperature, loads, etc. Entropy accumulates over cyclic loads. Via Equations (37) and (38), cyclic stress range $\sigma_{N}=\int_{t_{N}}^{t_{N+1}}\dot{\sigma }dt_{N}$ or $\dot{\sigma }=d\sigma_{N}/dt_{N}$ and cyclic strain range $\varepsilon_{N}=\int_{t_{N}}^{t_{N+1}}{\dot{\varepsilon }}_{e}dt_{N}+\int_{t_{N}}^{t_{N+1}}{\dot{\varepsilon }}_{p}dt_{N}$ together give the differential work density

(39) $$\delta W_{N}=\sigma_{N}:\left[d\varepsilon_{eN}+\left(\frac{1-n^{\prime }}{1+n^{\prime }}\right)d\varepsilon_{pN}\right].$$

Using Equation (38), boundary work done during time increment *dt* is

(40) $$\delta W=N_{dt}\delta W_{N}=N_{dt}\sigma_{N}:\left[d\varepsilon_{eN}+\left(\frac{1-n\prime }{1+n\prime }\right)d\varepsilon_{pN}\right].$$

Total strain accumulation over *dt* is

(41) $$\varepsilon =\int_{0}^{t}\left(d\varepsilon_{N}/dt_{N}\right)dt.$$

Dividing Equation (34) by volume V and combining with Equation (40) gives the change in Helmholtz energy density or toughness under high- or low-cycle fatigue loading. For stress-controlled loading, i.e., constant $\sigma_{N}$, and constant $N_{dt}$, Helmholtz energy dissipation density

(42) $$dA=-\left(\rho c\ln T+\frac{\alpha }{\kappa_{T}}\varepsilon \right)dT-N_{dt}\sigma_{N}:\left[d\varepsilon_{eN}+\left(\frac{1-n\prime }{1+n\prime }\right)d\varepsilon_{pN}\right]$$

and Helmholtz entropy generation density

(43) $$\delta S^{\prime }=-\left(\rho c\ln T+\frac{\alpha }{\kappa_{T}}\varepsilon \right)\frac{dT}{T}-N_{dt}\frac{\sigma_{N}}{T}:\left[d\varepsilon_{eN}+\left(\frac{1-n^{\prime }}{1+n^{\prime }}\right)d\varepsilon_{pN}\right]+\frac{{\sigma^{\prime }}_{f}:d({\sigma^{\prime }}_{f}/E)}{T}.$$

For strain-controlled loading, σ and ε are interchanged. When available, measurements of stress/strain response to loading should be used in place of Equations (35) and (36), which assume constant cyclic strain and strain energy. In Equation (43), the first term is the elemental microstructurothermal MST entropy density $\delta S{’}_{\mu T}$ characterizing internal material-dependent dissipation, the second is the boundary loading term $\delta S{’}_{W}$ characterizing energy dissipation across the system boundary via useful work output and environmental conditions, and the third is the reversible entropy $S{’}_{rev}$ defined using the component’s fatigue strength coefficient $\sigma \prime_{f}$. From Equation (42), MST energy density change $\delta A_{\mu T}=-\left(\rho c\ln T+\frac{\alpha }{\kappa_{T}}\varepsilon \right)dT$ and boundary work density $\delta A_{W}=-N_{dt}\sigma_{N}:\left[d\varepsilon_{eN}+\left(\frac{1-n\prime }{1+n\prime }\right)d\varepsilon_{pN}\right]$.

In renewable energy systems, the maximum work obtainable from a system, its Helmholtz free energy change $dA_{rev}$ or Gibbs free energy change $dG_{rev}$ may be defined cyclically. In all other systems $\int_{t_{0}}^{t}dA_{rev}dt=\Delta A_{rev}$ is constant and defined globally at manufacture as the maximum energy in the system or component from its newly manufactured state to full degradation, or locally just before onset of loading as the maximum energy change in the system/component before and after loading. This term is relatively inactive in the characteristic path-dependent evolution of entropy generation [58]. Neglecting the constant (between 2 states) reversible term in Equation (43) as in Prigogine et al.’s irreversible entropy generation formulations for active process/work interactions [42,43], phenomenological entropy generation or production in a mechanically loaded system is given as

(44) $$\delta {S^{\prime }}_{phen}=-\left(\rho c\ln T+\frac{\alpha }{\kappa_{T}}\varepsilon \right)\frac{dT}{T}-N_{dt}\frac{\sigma_{N}}{T}:\left[d\varepsilon_{eN}+\left(\frac{1-n^{\prime }}{1+n^{\prime }}\right)d\varepsilon_{pN}\right].$$

The above considers a loading rate *h* different from sampling rate 1/*dt*. If cyclic loading and data sampling rates are the same, $N_{dt}=1$. Similar expressions can be obtained for shear stress τ and shear strain γ, for torsion.

#### 4.3.2. Infinite Life Design

In infinite life design, loading and material behavior are predominantly in the elastic region, hence elastic formulations are reliable [4,5,6]. The Wohler (S-N) curve and the Goodman diagram show the region below the fatigue limit in which certain materials may be loaded indefinitely without failure. Others such as the Soderbeg criteria are based on the component’s elastic response. For bending, normal strain $\varepsilon_{e}=\frac{\sigma }{E}$. For torsion, shear strain $\gamma_{e}=\frac{\tau }{G}$. For simultaneous loads such as combined bending and torsion, von Mises formulations can be used. Predominant elastic interactions are nearly isothermal, so the Helmholtz energy density change from Equation (42) with $d\varepsilon_{pN}=0$ becomes

(45) $$dA=N_{dt}\left(\sigma_{N}:d\varepsilon_{eN}\right),$$

and phenomenological Helmholtz entropy generation density from Equation (44)

(46) $$\delta {S^{\prime }}_{phen}=-\frac{1}{T}N_{dt}\left(\sigma_{N}:d\varepsilon_{eN}\right).$$

Equation (46) is the minimum entropy generation in a dynamically loaded system (in terms of stress and strain) defined by Prigogine’s stationary non-equilibrium theorem [43]. At the reversibility limit or for a fully reversible (elastic) system—which would imply a “true” infinite life design—boundary temperature T is constant, giving uniform $\delta {S^{\prime }}_{phen}$. Metals such as steel exhibit nearly reversible characteristics (infinite life) when loaded below fatigue limits [2,3,4,5,6,7]. Equation (46) also applies to isothermal loading conditions.

### 4.4. Degradation-Entropy Generation (DEG) Analysis

Rewriting Equations (23) and (24) in rate form without the compositional change term, and integrating over time gives the total change in Helmholtz energy from $t_{0}$ to *t* as $\Delta A=-\int_{t_{0}}^{t}S\dot{T}dt-\int_{t_{0}}^{t}Y\dot{X}dt$, and phenomenological entropy generation as

(47) $${S^{\prime }}_{phen}=-\int_{t_{0}}^{t}\frac{S\dot{T}}{T}dt-\int_{t_{0}}^{t}\frac{Y\dot{X}}{T}dt.$$

Via the DEG formulations in Section 2, system degradation measured by fatigue parameter w is directly related to phenomenological entropy generation as

(48) $$w=B_{\mu T}\int_{t_{0}}^{t}-\frac{S\dot{T}}{T}dt+B_{W}\int_{t_{0}}^{t}-\frac{Y\dot{X}}{T}dt=B_{\mu T}S{’}_{\mu T}+B_{W}S{’}_{W}.$$

Via Equation (12), DEG coefficients

(49) $$B_{\mu T}=\frac{\partial w}{\partial S\prime_{\mu T}};\;B_{W}=\frac{\partial w}{\partial S\prime_{W}}$$

which pertain to MST entropy $S{’}_{\mu T}=\int^{\text{​}}-\frac{S\dot{T}}{T}dt$ and boundary work entropy $S{’}_{W}=\int^{\text{​}}-\frac{Y\dot{X}}{T}dt$, respectively, can be evaluated from measurements of slopes of w versus entropy production components $S{’}_{i}$.

#### 4.4.1. Applying the Degradation-Entropy Generation Theorem to Cumulative Strain (or Stress)

Assuming the cyclic effects of measured strain are cumulative (to account for all simultaneous variable and complex loading) and vary with strain intensity, a strain measure may be defined for the DEG theorem (using Equation (43) for $S{’}_{phen}$) as

(50) $$\varepsilon =\int_{t_{0}}^{t}\dot{\varepsilon }dt=-B_{\mu T}\int_{t_{0}}^{t}\left(C\ln T+\frac{\varepsilon \alpha }{\kappa_{T}}\right)\frac{\dot{T}}{T}dt+B_{W}\int_{t_{0}}^{t}N_{dt}\frac{\sigma_{N}}{T}:\left[{\dot{\varepsilon }}_{eN}+\left(\frac{1-n^{\prime }}{1+n^{\prime }}\right){\dot{\varepsilon }}_{pN}\right]dt.$$

For truly infinite life and assuming elastic work

(51) $$\varepsilon =B_{W_{e}}\frac{\sigma_{N}}{T}\varepsilon_{e}\;.$$

If loading is strain-controlled, the measured stress response may become a cumulative degradation measure and similar relations developed.

## 5. Fatigue Experiments and Data Analysis—Instantaneous Characterization

**Figure 1:**
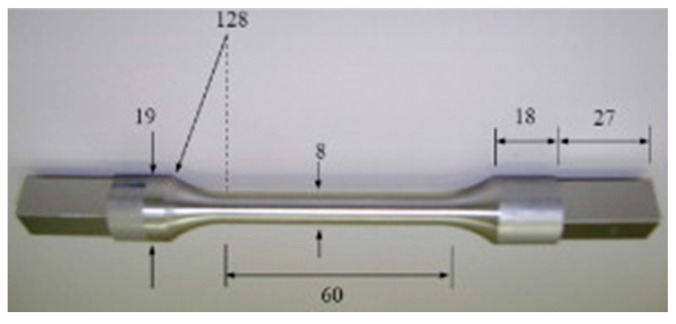
Torsion fatigue-tested steel sample SS 304 showing dimensions in mm, reproduced from [14].

Low-cycle fatigue data by Naderi, Amiri and Khonsari [15,18] will verify formulations. Details about equipment, procedures and data are in references [15,18]. Briefly, at sampling frequency 7.5 Hz, a high-resolution infra-red camera monitored temperature profiles of the SS 304 stainless steel fatigue specimen depicted in Figure 1, with material properties in Table 1. 

Displacement-controlled bending and torsional loads oscillated at 10 Hz. Plots in the upcoming figures, generated from Naderi et al.’s data, have “*a*” subfigures on the left pertaining to bending fatigue, and “*b*” subfigures on the right pertaining to torsional fatigue. Signs follow the thermodynamic convention of the formulations, e.g., boundary loading and MST energies and entropies are negative.

**Figure 2:**
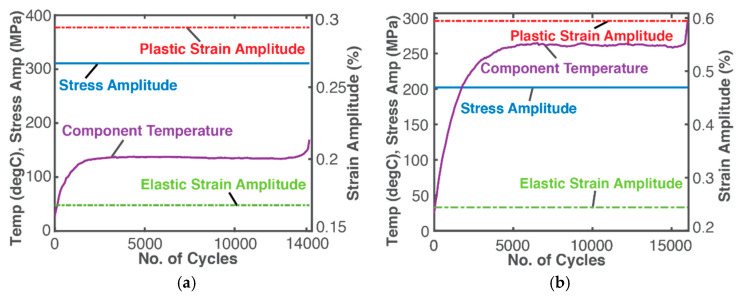
Parameters during cyclic (**a**) bending and (**b**) torsional fatigue of the SS 304 steel at a constant frequency 10 Hz and displacement loading δ = 45.72 mm and δ = 33.02 mm [15]. Temperatures and cyclic stress amplitude are on the left axis, and cyclic strain amplitude is on the right.

Figure 2a plots the constant cyclic stress amplitude obtained from $\sigma_{a}=\sigma \prime_{f}{\left(2N_{f}\right)}^{b}$, constant elastic strain amplitude from Hooke’s law $\varepsilon_{ea}=\frac{\sigma_{a}}{E}$, constant plastic strain amplitude from Morrow’s relation [53] $\varepsilon_{pa}=\varepsilon \prime_{f}{\left(\frac{\sigma_{a}}{\sigma \prime_{f}}\right)}^{1/n\prime }$ and measured temperature *T* versus number of cycles *N*. Torsional loading in part (b) of the figures employs shear stress τ and shear strain γ. In the rest of this article σ and ε will denote generalized stress and strain. Number of cycles accumulated at failure was $N_{f}$ = 14,160 for bending, $N_{f}$ = 16,010 for torsion [15].

For bending, Figure 2a shows a constant normal stress amplitude $\sigma_{a}$ = 311 MPa, a steady normal elastic strain amplitude $\varepsilon_{ea}$ = 0.17% and steady normal plastic strain amplitude $\varepsilon_{pa}$ = 0.29%. For torsion, Figure 2b shows a constant shear stress amplitude $\tau_{a}$ = 202 MPa, a steady elastic shear strain amplitude $\gamma_{ea}$ = 0.24% and steady shear plastic strain amplitude $\gamma_{pa}$ = 0.59% (this last value is high due to the high torsional fatigue ductility coefficient $\gamma \prime_{f}$ found in literature, see Table 1). In both cases, a steep rise in temperature (purple curves in Figure 2) arose from high hysteresis dissipation from an initial rest state. After this initially transient response region (about 2000 cycles for bending and 5000 for torsion), pseudo-steady state temperature persists until a sudden rise occurs, followed by fatigue failure [14,15]. Substituting Naderi et al.’s data into Equations (42), (43) and (50), Table 2 was constructed. Units of %*N*, GJ/m^3^ and MPa/K are used for cumulative strain, energy density and entropy density respectively (1 GPa = 1 GJ/m^3^; 1 MPa/K = 1 MJ/m^3^K) giving strain-based *B* coefficient units of %*N*K/MPa.

**Figure 3:**
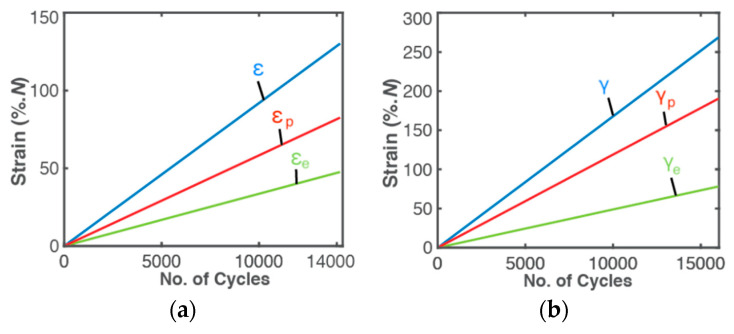
Cumulative strains—elastic (green), plastic (red) and total (blue) vs number of load cycles *N* for (**a**) bending—normal strain ε; (**b**) torsion—shear strain.

Table 2 column 1 lists fatigue loading types, bending and torsion. Section 4 formulations involved integrals over time. Trapezoidal quadratures with widths inverse to the data sampling frequency (7.5 Hz [15]) estimated time integrals. For a process occurring from $t_{0}$ to t, cumulative strain in Equation (41), Table 2 column 2, was estimated as

(52) $$\varepsilon =\int_{0}^{t}\dot{\varepsilon }dt=\int_{t_{0}}^{t}\left(d\varepsilon_{N}/dt_{N}\right)dt\approx \left(\frac{1}{\Delta t_{N}}\right)\overset{m}{\underset{1}{\sum }}\left(\varepsilon_{m}\right)\Delta t=N_{\Delta t}\overset{m}{\underset{1}{\sum }}\left(\varepsilon_{m}\right)$$

where indices 1, 2, 3, …, *m* correspond to times $t_{1}$,$\;t_{2}$,$\;t_{3}$, …, $t_{m}$ and $\Delta t=t_{m}-t_{m-1}$, period $\Delta t_{N}=1/10$ [15], data sampling time increment Δt=1/7.5, and total number of cycles within sampling time increment $N_{\Delta t}=10/7.5$, see Equation (38). Finally, $\varepsilon_{m}$ is strain range at $t_{m}$. Shear strain γ was similarly obtained for torsion. Via constant cyclic strain ranges [53] $\varepsilon_{N}$ and $\gamma_{N}$, cumulative strains varied linearly with number of cycles *N* until sudden failure, with no indication of failure onset (Figure 3).

### 5.1. Instantaneous Evolution of Helmholtz Energy Density (Toughness) and Entropy Density

**Figure 4:**
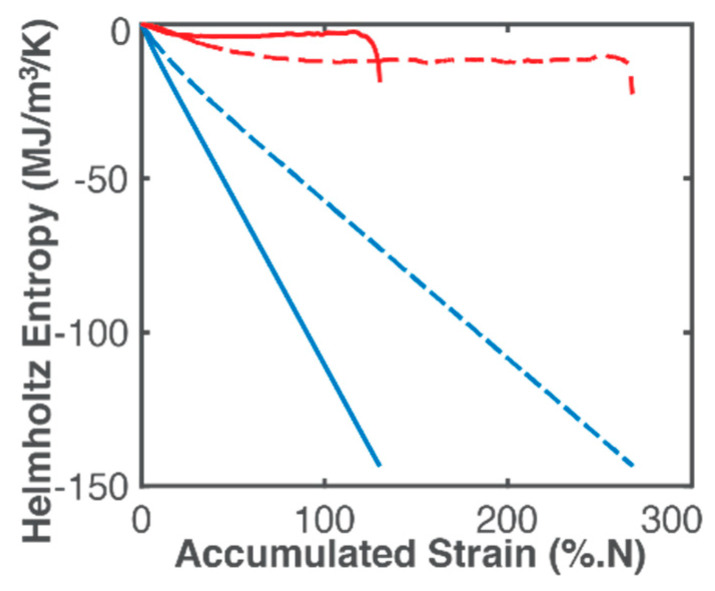
Phenomenological Helmholtz entropy density components—load entropy (blue plots) and MST entropy (red plots)—versus accumulated strain during bending (continuous curves) and torsion loading (dashed curves). Note 1 MJ/m^3^/K = 1 MPa/K.

Table 2 lists components of Helmholtz toughness, Equation (40), $A_{W}=-N_{\Delta t}\sum_{1}^{m}\left(\sigma_{m}\left[\varepsilon_{em}+\varepsilon_{pm}\left(\frac{1-n\prime }{1+n\prime }\right)\right]\right)$ (column 3) and $A_{\mu T}=-\sum_{1}^{m}\left(\rho c\ln T_{m}+\frac{\alpha }{\kappa_{T}}\varepsilon_{m}\right)\Delta T_{m}$ (column 4) during bending and torsional fatigue of the steel member. Figure 4 plots the accumulated boundary/load (blue curves) and MST (red curves) entropy densities. In Figure 4, a near linear relationship is observed between load entropy, column 5 of Table 2,

(53) $$S\prime_{W}=\int_{0}^{t}\frac{\sigma \dot{\varepsilon }}{T}dt=N_{\Delta t}\overset{m}{\underset{1}{\sum }}\left\{\frac{\sigma_{m}}{T_{m}}\left[\varepsilon_{em}+\varepsilon_{pm}\left(\frac{1-n\prime }{1+n\prime }\right)\right]\right\}$$

and accumulated strain for the assumed constant stress amplitude loading and constant strain amplitude response, with a slight curvature from the initial temperature rise (Figure 4). Table 2 shows the same failure value of 143.5 MPa/K for both bending and torsion, as previously observed by Naderi, Amiri and Khonsari [15,18,19,20], unlike load (strain) energy density $A_{W}$. MST entropy density (red curves), column 6,

(54) $$S\prime_{\mu T}=\int_{0}^{t}-\left(\rho c\ln T+\frac{\alpha }{\kappa_{T}}\varepsilon \right)\frac{\dot{T}}{T}dt=-\overset{m}{\underset{1}{\sum }}\left(\rho c\ln T_{m}+\frac{\alpha }{\kappa_{T}}\varepsilon_{m}\right)\frac{\Delta T_{m}}{T_{m}}$$

shows a profile significantly influenced by the measured temperature profile but less steep than the latter due to the microstructural effect (second right side term in Equation (54), see Figure 4). Accurate determination of MST entropy includes effects of instantaneous temperature, especially for anisothermal conditions. Amiri and Khonsari [14] related fatigue life to the gradient of the initial temperature rise. Both MST energy and entropy densities are higher for torsion than bending. At every instant, load entropy $S\prime_{W}$ and an accompanying MST entropy $S\prime_{\mu T}$ are produced, both at the instantaneous boundary temperature. Figure 4 shows that with $S\prime_{\mu T}$ stabilizing with steady temperature, $S\prime_{W}$ quickly becomes more significant to total irreversible entropy, a desired feature (the boundary loading is the component’s output work, hence the higher its contribution to total phenomenological entropy, the more optimal the component’s response to loading). However, the sudden rise in magnitude of $S\prime_{\mu T}$ just before failure is not evident in load (boundary work) entropy.

**Figure 5:**
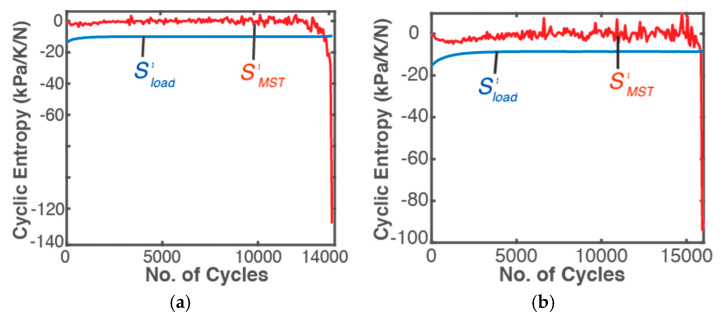
Cyclic phenomenological entropy generation components—load (blue) and MST (red) entropies—versus number of cycles *N* for (**a**) bending, (**b**) torsion of the SS 304 steel specimen.

Figure 5 plots rates of phenomenological Helmholtz entropy generation components—load and MST entropies—versus number of cycles. Cyclic load entropy (blue curves) starts at a slightly higher rate and quickly steadies as quasi-steady temperature is reached. MST entropy rate (red curves in Figure 5, right axes label) shows more significant fluctuations with sudden discontinuity (large spike) just before failure. With measured non-constant strain response using appropriate equipment (particularly for variable and complex load types), the boundary work/load entropy characteristics could differ from those presented here in which constant stress and strain amplitudes were used, as often done in fatigue analysis [15,53,54,55].

### 5.2. DEG Analysis—Strain Versus Entropy (Linear Transformation)

**Figure 6:**
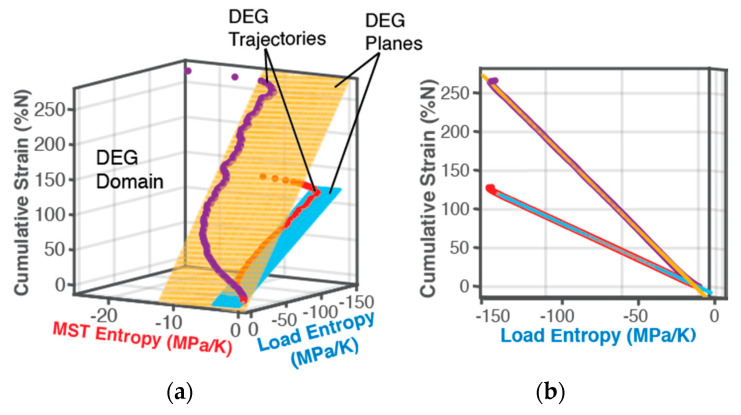
3D plots and linear surface fits of cumulative strain vs load entropy and MST entropy during cyclic bending (red points, blue plane) and torsion (purple points, orange plane) of SS 304 steel sample, showing in (**b**) a goodness of fit of R^2^ = 1, indicating a linear dependence on the 2 active processes. In (**a**), loading trajectories start from lowest corner. (Axes are not to scale and colors are for visual purposes only).

By associating data from various time instants, accumulated strain ε from Equation (41) was plotted versus accumulated entropies $S\prime_{W}$ and $S\prime_{\mu T}$ in 3-dimensional Figure 6. Time is a parameter along curves: successive points from bottom to top on each curve correspond to later times along the fatigue evolution. Coincidence of measured data points with planar surfaces in Figure 6 has goodness of fit $R^{2}$ = 1, asserting a statistically perfect fit for all cases prior to impending failure. The end views emphasize the coincidence of points with the planes. This suggests a linear dependence of degradation/fatigue on both the actual output work/boundary loading and MST entropies at every instant of loading. The measured data points in the curves of Figure 6 that define the component’s paths during loading—its Degradation-Entropy Generation (DEG) trajectories—lie on planar DEG surfaces. The orthogonal 3D space occupied by the DEG surfaces, the component’s material-dependent DEG domain, appears to characterize the allowable regime in which the component can be loaded.

The dimensions of the DEG planes are determined by the accumulation of the entropy generation components before failure onset. As previously observed, bending and torsion have the same boundary work entropy dimension, indicating that this dimension is characteristic of the specimen material, not the process, further verifying Naderi, Amiri and Khonsari [15,18,19,20]. Overall, $A_{W}$ and $S\prime_{W}$ are about 7 (6 for torsion) times $A_{\mu T}$ and $S\prime_{\mu T}$, respectively. MicroStructuroThermal (MST) dissipation accompanies boundary interaction/loading. Figure 6b also shows points of the trajectory not lying on the DEG plane. These points violate the linearity of Equation (50), suggesting another fundamentally different dissipative process at work. The pseudo-constant temperature region (see Figure 2) appears in the DEG domain as a pseudo-constant MST region, with fluctuations.

*Degradation Coefficients*$B_{i}$: Degradation coefficients $B_{W}$ and $B_{\mu T}$, partial derivatives of fatigue measure—cumulative strain—with respect to loading and MST entropies respectively, Equation (49), were estimated from the orientations of the surfaces in Figure 6, see columns 7 and 8 of Table 2. For bending, $B_{W}=-0.92$ %K/MPa and $B_{\mu T}=0.22$ %K/MPa, and for torsion, $B_{W}=-1.96$ %K/MPa and $B_{\mu T}=0.42$ %K/MPa. A lower value for B implies lesser impact on fatigue degradation.

### 5.3. Phenomenological Transformation Versus Measured/Estimated Fatigue Parameter

**Figure 7:**
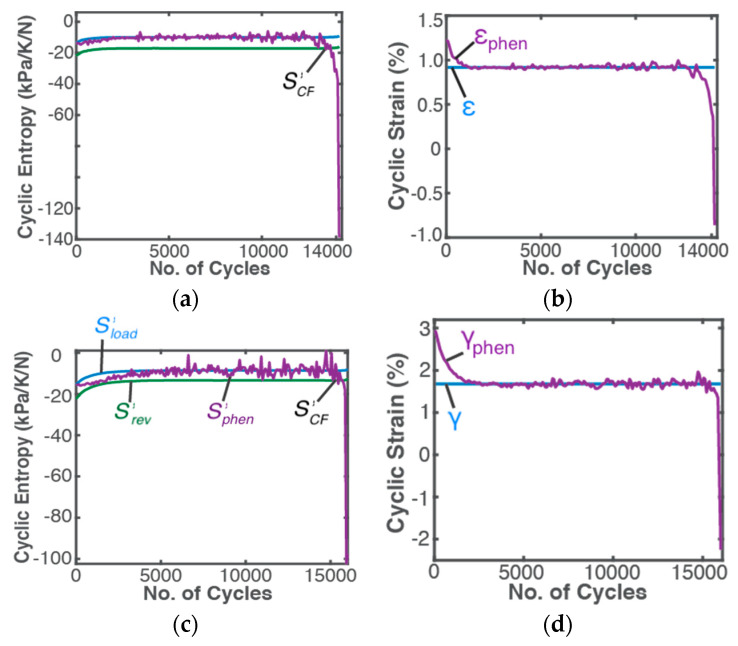
Cyclic entropy generation—load (blue), phenomenological (purple) and reversible (green)—as well as corresponding cyclic strain—estimated constant (blue) and phenomenological (purple)—during bending (**a**,**b**) and torsion (**c**,**d**) of the steel specimen. Region between *S’_phen_* and *S’_rev_* is entropy generation *S’* given by Equation (25). A similar critical failure entropy S’_CF_ is shown for both loading types.

Using constant *B* coefficients given in Table 2, instantaneous entropy transformations were projected onto the estimated fatigue or degradation parameter to determine phenomenological fatigue parameter, analogous to the previously defined phenomenological entropy generation. Figure 7a,c show reversible Helmholtz entropy $S\prime_{rev}$ (green curves), phenomenological entropy $S\prime_{phen}$ (purple curves) and boundary work/load entropy $S\prime_{W}$ (blue curves) during bending and torsion of the steel sample. In Figure 7b,d, DEG-evaluated phenomenological strains $\varepsilon_{phen}$ and $\gamma_{phen}$ (purple curves) and estimated strains $\varepsilon =\varepsilon_{e}+\varepsilon_{p}$ and $\gamma =\gamma_{e}+\gamma_{p}$ (blue curves) are plotted. The actual transient response of the component under load is unobservable in cyclic strains ε and γ estimated from currently available LCF analysis methods. The DEG methodology, via entropy which uses a component’s instantaneous temperature, introduces more representative cyclic strains $\varepsilon_{phen}$ and $\gamma_{phen}$ which consistently show all instantaneous nonlinear transitions during loading including the initially high energy dissipation rate observable in Figure 7b,d.

Substituting coefficient values into Equation (48) gives the SS 304 steel sample’s DEG cumulative strain-based fatigue life/degradation models for bending and torsion

(55) $$\varepsilon_{phen}=\left(0.22S{’}_{\mu T}-0.92S{’}_{W}\right)*10^{-6}$$

(56) $$\gamma_{phen}=\left(0.42S{’}_{\mu T}-1.96S{’}_{W}\right)*10^{-6},$$

which linearly relate the phenomenological fatigue strains $\varepsilon_{phen}$ and $\gamma_{phen}$ to the phenomenological entropies $S{’}_{phen}=S{’}_{W}+S{’}_{\mu T}$ produced. Via the known relations between entropy production and the active variables of loads, materials and environment, Equations (55) and (56), in turn, relate the fatigue strains to the phenomenological variables.

#### Critical Failure Entropy $S\prime_{\mathrm{CF}}$—MST Entropy and Fatigue Failure

A corollary of the DEG theorem: “if a critical value of degradation measure at which failure occurs exists, there must also exist critical values of accumulated irreversible entropies” [24]. Naderi, Amiri and Khonsari’s extensive measurements [15,18,19,20] showed existence of a material-dependent fatigue fracture entropy *FFE* or $S\prime_{f}$ evaluated as the load entropy (using constant plastic strain amplitude) accumulated at failure. The data of this article, obtained from references [15,18], verified similar magnitudes of cumulative $S\prime_{W}$ for both bending and torsion of the SS 304 steel specimen. To anticipate onset of failure, Khonsari et al. empirically determined a normalized onset of failure entropy criterion $\frac{S\prime }{S\prime_{f}}\leq 0.9$ from several temperature profiles measured during loading [17]. Other common fatigue tools like σ—N and ε—N curves, with constant stress and strain amplitudes, do not exhibit the critical phenomenon. The DEG domain shows a distinct and consistent critical onset of failure. In Figure 7a,c, the abrupt drop in phenomenological Helmholtz entropy generation just before failure is attributed to the sudden rise in specimen temperature. Via the *B* coefficients, this abrupt drop is transferred to phenomenological strain, Figure 7b,d, introducing the critical feature to the hitherto steady fatigue measure, cumulative strain.

To understand the entropy generation critical value, reexamine Figure 7. The region between the reversible entropy $S\prime_{rev}$ and phenomenological entropy $S_{phen}$ curves—the subtraction difference—is entropy generation. With the stable evolution criterion $S_{rev}\leq S_{phen}<0$, the abrupt spike in $S_{phen}$ resulted in the second law-prohibited negative entropy generation of Equations (25) and (43). The intersection of $S_{rev}$ and $S_{phen}$ marks the critical failure entropy $S\prime_{CF}$ (Figure 7a,c). With constant cyclic stress and strain, the cyclic load entropy (blue plots in Figure 7a,c) trends directly with measured temperature (Figure 2), accumulating linearly over time (Figure 4). Comparing Figure 5 and Figure 7 shows that the downward spike in cyclic $S_{phen}$, also observed as the trajectory discontinuity in the DEG domains, is introduced by the microstructurothermal (MST) entropy composed of a thermal change- and microstructural change-induced internal entropy generation. If a pseudo-steady temperature was not attained, the MST entropies would have risen continuously and accelerated failures. Note that the initial temperature rise is less for bending fatigue than torsion [15], Figure 2, the effect of which is evident in the MST dimensions of the respective DEG planes. Hence, MST entropy measures a component’s instantaneous instabilities and ultimate failure. In other forms of loading including thermal and chemical cycling of components, the significance of MST entropy is underscored by the limited safe operating temperature ranges specified by device manufacturers to prevent instabilities/runaway events.

### 5.4. Nonlinear Response

Via Morrow [53] and Lemaitre and Chaboche [7], this article assumed a constant cyclic strain response to constant stress loading, similar to Khonsari et al. However, for variable and complex asynchronous loading, a nonlinear response is typically observed.

## 6. Discussion and Contributions

**Figure 8:**
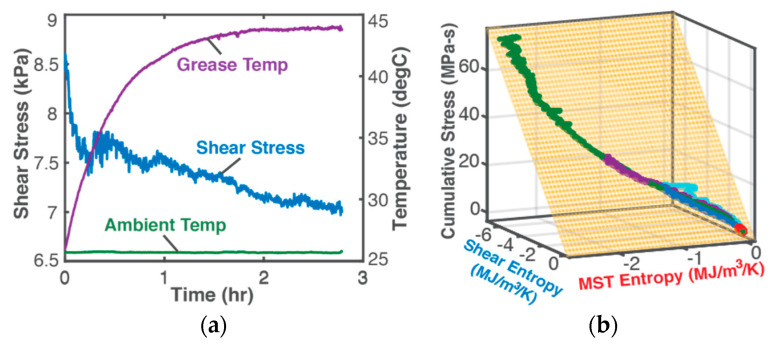
(**a**) Monitored parameters—shear stress and temperatures—and (**b**) DEG domain for mechanical shearing of high-consistency lubricant grease show multiple nonlinear shear stress trajectories coincident with the same DEG plane. Reproduced from [32].

Other experimental verification of the DEG methodology include nonlinear shear stress response to shear rate-controlled shearing of lubricant grease [32], (Figure 8), and abusive cycling of Li-ion batteries [40] have been demonstrated by Osara and Bryant. In Figure 8b, the DEG trajectories—independent datasets measured at different times and durations—all lie on the same DEG plane, characteristic of the grease.

Similar to Prigogine’s successful extension of hitherto reversible thermodynamic formulations to irreversible and non-equilibrium processes and states [35,36,42,43], this study derived and verified a consistent utility-based, time-dependent system entropy generation. Based on Gibbs theory of thermodynamic stability of equilibrium states and the second law entropy balance, this article demonstrated that

- phenomenological entropy generation $S\prime_{phen}$ is the sum of boundary work/load entropy $S\prime_{W}$ and microstructurothermal MST entropy $S\prime_{MST}$;
- entropy generation is the difference between phenomenological $S\prime_{phen}$ and reversible $S\prime_{rev}$ Helmholtz entropies at every instant;
- entropy generation is always non-negative in accordance with the second law, whereas components $S\prime_{phen}$ and $S\prime_{rev}$ are directional, negative for a loaded system. This implies $\left|{S^{\prime }}_{phen}\right|<\left|{S^{\prime }}_{rev}\right|$ during load application in accordance with experience and thermodynamic laws. The actual work obtained from the system is always less than the maximum/reversible work.

Stress and strain (bending and torsional) were used as system conjugate variables to characterize energy dissipation and entropy generation in a loaded metal bar.

### 6.1. Features of the DEG Methodology

Basaran et al. [9,10,11,12,13] and Khonsari et al. [14,15,16,17,18,19,20,21,22,23] in several fatigue-entropy works demonstrated the robustness, consistency and ease of use of entropy generation-based damage/fatigue analysis. This article showed that the DEG methodology relates accumulated irreversibilities to the resulting damage in systems using entropy generation components. DEG theorem methods can accurately describe a system’s fatigue level during operation in a fatigue measure versus entropy generation components space. Since the entropy generation depends on the load, materials and environment, the DEG methods in turn relate a material’s fatigue measure to the working phenomenological variables of interest.

#### 6.1.1. DEG Trajectories, Surfaces and Domains

Thermodynamics authors have consistently used multi-dimensional orthogonal spaces to describe thermodynamic states of reversible processes: Callen’s thermodynamic configuration space [36], Messerle’s energy surface [47] and Burghardt’s equilibrium surface [41]. This study introduced the DEG domain, a multi-dimensional space that linearly characterizes a real system’s nonlinear phenomenological transformation paths. Proper formulation of the governing entropies from the active dissipative processes is required to accurately determine fatigue degradation during loading.

DEG trajectories characterize loading conditions (torsion, bending, stress/strain amplitudes, etc.); DEG surfaces appear to characterize component material and process rates; and the DEG domain seems to define the normal operating/aging region and the failure region, fully characterizing the component’s life for all loads and process rates. A component having a DEG domain with large accumulated fatigue measure span and small MST entropy span (relative to load entropy dimension) will accumulate more load strain (or do more work) before failure. Hence, the DEG fatigue methodology can directly compare designs and materials for manufacture and applications.

The out-of-plane points at the termini of the DEG trajectories of Figure 6 occurred at the onset of failure. Here, a crack in the fatigued specimen attains a critical length, which causes a catastrophic fracture crack growth that ruptures the specimen [56,59]. Fracture cracking as opposed to fatigue cracking involves fundamentally different dissipative processes and entropy generation [60]. The DEG model could add this effect via an additional term in Equation (50) for fracture entropy generation, similar to the fracture entropy formulated by Rice [61]. This third orthogonal entropy generation axis in Figure 6 would extend the plots to 4D: cumulative strain vs. load entropy, MST entropy and fracture entropy. Via the thermodynamic state principle [45] and the DEG theorem, other concurrent independent processes would append additional dimensions to the DEG domain.

#### 6.1.2. DEG Coefficients

Unlike existing fatigue methods wherein stress-life and strain-life diagrams predict suitability of a component using extensive data from several failed samples, DEG coefficients can be obtained from one or two representative samples and applied to other components of the same material(s) undergoing similar processes. These coefficients show a component’s response to prevalent interactions and conditions by quantifying the processes’ contributions to fatigue failure.

Boundary work/load coefficient $B_{W}$ is negative for positive evolution of fatigue measure—load entropy is negative during loading. MST coefficient $B_{\mu T}$ has varying sign characteristic. To understand $B_{\mu T}$ sign changes, rewrite Equation (48) as $w=w_{phen}=B_{\mu T}S\prime_{\mu T}+B_{W}S\prime_{W}$ and rearrange to get

(57) $$B_{\mu T}=\frac{1}{S\prime_{\mu T}}\left(w_{phen}-B_{W}S\prime_{W}\right)$$

where phenomenological fatigue measure $w_{phen}$ (e.g., $\varepsilon_{phen})$ fluctuates about the load-based measure $B_{W}S\prime_{W}$ (Figure 7b,d), making the parenthesis expression in Equation (57) fluctuate about zero during operation. It is also observed from Figure 5 that instantaneous MST entropy $S\prime_{\mu T}$ fluctuates about zero (more significantly for torsion).

### 6.2. Entropy Generation vs Number of Cycles—A Linear Arrow of Time

**Figure 9:**
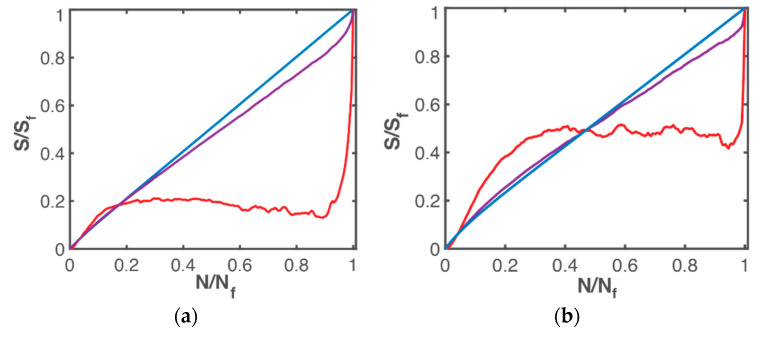
Normalized phenomenological entropy and components—load (blue), MST (red), phenomenological (purple) versus normalized cycles for (**a**) bending, (**b**) torsion of the SS 304 steel specimen.

Describing entropy *S* as “time’s arrow”, Eddington [36] stated

(58) $$\mathrm{at}\;t\geq t_{0},\;S\geq S_{0}$$

for an isolated system where is entropy at initial/reference time $t_{0}$. Amiri et al. [15,18,23] via several experiments, observed an approximately linear relationship between normalized entropy and number of cycles. In Figure 9, normalized load entropy $S\prime_{W}/S\prime_{Wf}$ (blue curves), microstructurothermal (MST) entropy $S\prime_{\mu T}/S\prime_{\mu Tf}$ (red curves) and total phenomenological Helmholtz entropy generation $S\prime_{phen}/S\prime_{phen,f}$ (purple curves) vs. normalized number of cycles $N/N_{f}$ are presented for bending fatigue (9a) and torsional fatigue (Figure 9b). An approximate linearity was observed in $S\prime_{W}/S\prime_{Wf}$. Figure 9 also shows that $S\prime_{\mu T}/S\prime_{\mu Tf}$ and, consequently, $S\prime_{phen}/S\prime_{phen,f}$ do not evolve linearly with $N/N_{f}$; entropy generation as prescribed by the Helmholtz formulation for stress-strain loading, Equation (43), for an anisothermal process, includes a significant nonlinear microstructurothermal (MST) component.

**Figure 10:**
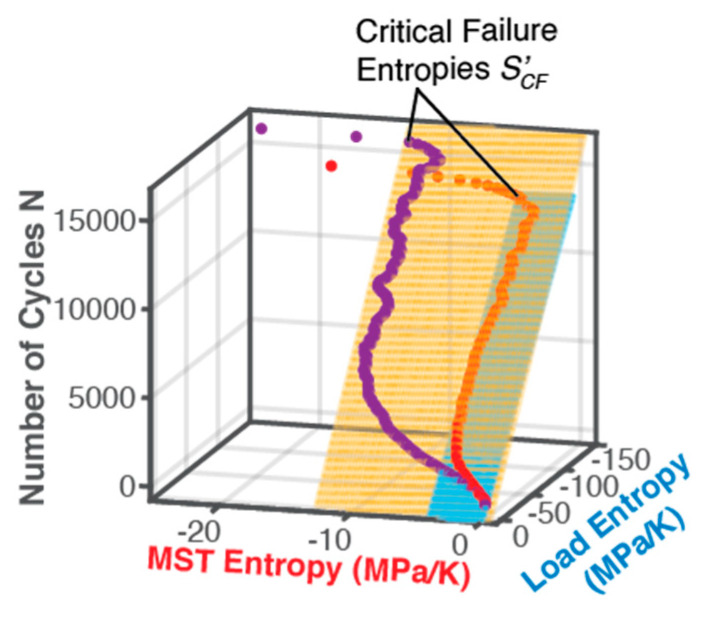
3D plots and linear surface fits of numbers of cycles N vs load entropies and MST entropies during cyclic bending (red points, blue plane) and torsion (purple points, orange plane) of the SS 304 sample. Life trajectories start from lowest corner. (Axes are not to scale and colors are for visual purposes only).

Similar to Figure 6 which uses accumulated strain for component characterization via the DEG methodology, Figure 10 plots the components of phenomenological entropy generation $S\prime_{W}$ and $S\prime_{\mu T}$ versus number of cycles N. Via Equation (38), N can be replaced by time *t* via $\int_{t_{0}}^{t_{f}}dt=\int_{N_{0}}^{N_{f}}\frac{dN}{h}$ where h is the load cycle frequency. For constant h and $N_{0}\left(t_{0}=0\right)=0,\;t=\frac{N}{h}$. Considering the SS 304 steel torsional fatigue (last row of Table 2 and (b) figures in article), $N_{f}=16010$ and h=10 Hz give total time to failure $\Delta t_{f}$ = 26.68 min. As depicted by Figure 10 using number of cycles, the DEG methodology linearizes the natural evolution of entropy generation over time: there exists a *linear* arrow of time.

Recall Equation (48) with $t_{f}$ = w:

(59) $$t_{f}=B_{\mu Tt}S\prime_{\mu T}+B_{Wt}S\prime_{W}.$$

From orientations of the Figure 10 DEG planes with $t_{f}$ = $N_{f}/h$, $B_{\mu Tt}=12.31$ NK/MPa and $B_{Wt}=-99.78$ NK/MPa for bending. Equation (59) linearly relates entropy generation to degradation time, cycle life or time to failure for components under all types of load. Therefore, with a consistent evolution criterion, *entropy generation* via *the DEG theorem is a linear arrow of time*, Equation (59) and Figure 10. With DEG domains such as Figure 10, all systems undergoing cyclic or time-dependent loading can be fully and instantaneously characterized based on degradation or failure time $t_{f}$. The horizontal axes dimensions of the DEG domain (values of $S\prime_{W}$ and $S\prime_{\mu T}$ at $N_{f}$) can be directly correlated with other existing fatigue analysis methods that use $N_{f}$ like the common σ—N and ε—N curves. The DEG approach appears universal and can be directly adapted to state of health and performance monitoring. The results in this article show that the DEG method can anticipate and potentially monitor and prevent fatigue failures accurately.

## 7. Summary and Conclusions

Fundamental irreversible thermodynamics and the degradation-entropy generation DEG theorem were applied to fatigue. The DEG theorem’s fatigue/degradation model, which related a strain measure of fatigue to the load (boundary work) and MicroStructuroThermal entropies produced, was formulated and verified. A thermodynamic potential, the Helmholtz free energy, replaced steady state assumptions of previous DEG applications and employed the instantaneously applicable first and second laws of thermodynamics. The significance of the MicroStructuroThermal MST entropy and reversible Helmholtz entropy to total entropy generation and fatigue failure was demonstrated. Plots—DEG domains, Figure 6, Figure 8 and Figure 10—derived from published experimental data [15,18] showed the DEG-predicted linearity between fatigue/life measures and entropy generation components with goodness of fit R^2^ = 1. Flexibility of fatigue parameter selection was also demonstrated. The DEG theorem provides a structured approach to component/system fatigue/degradation modeling, removing the need for many measurements, numerous curve fits and multiple analysis tools.

## Figures and Tables

**Table 1:** Material properties for SS 304 steel used in evaluating loading parameters [2,15,55,56].

Property	Bending	Torsion
Modulus, GPa	*E* = 195	*G* = 82.8
Fatigue strength coefficient, MPa	$\sigma \prime_{f}$ = 1000	$\tau \prime_{f}$ = 709
Fatigue strength exponent *b*	−0.114	−0.121
Fatigue ductility coefficient	$\varepsilon \prime_{f}$ = 0.171	$\gamma \prime_{f}$ = 0.413
Fatigue ductility exponent *c*	−0.402	−0.353
Cyclic strain hardening exponent *n’*	0.287	0.296
Specific heat capacity C, J/kg K	500	
Density ρ, kg/m^3^	7900	
Coefficient of linear thermal expansion α	17.3 × 10^−6^	

**Table 2:** Helmholtz energy-based DEG fatigue analysis results for bending and torsional loading to failure of the SS 304 steel specimen in Figure 1.

Load	$\varepsilon_{f},\gamma_{f}$ %*N*	$A_{W}$ GJ/m^3^	$A_{\mu T}$ GJ/m^3^	$S\prime {}_{W}$ MPa/K	$S\prime {}_{\mu T}$ MPa/K	$B_{W}$ %*N*K/MPa	$B_{\mu T}$ %*N*K/MPa
Bending	130.1	−58.0	−7.8	−143.5	−18.8	−0.92	0.22
Torsion	268.5	−73.4	−12.3	−143.5	−24.1	−1.96	0.42

## Abbreviations

**Nomenclature**	**Name**	**Unit**
*A*	Helmholtz free energy density	J/m^3^
*B*	DEG coefficient	%NK/MPa
*C*	heat capacity	J/K
*N*	number of cycles	
*N*, *N_k_*	number of moles of substance	mol
*P*	dissipative process energy	J
*P*	pressure	Pa
*Q*	heat	J
*S*	entropy density or entropy content	J/m^3^K or MPa/K
*S’*	entropy generation or production	J/m^3^K or MPa/K
*t*	time	s
*T*	temperature	degC or K
*U*	internal energy	J
V	volume	m^3^
*w*	degradation measure	
*W*	work, strain energy density	J, J/m^3^
**Symbols**		
*α*	thermal expansion coefficient	/K
*κ_T_*	isothermal loadability	
*μ*	chemical potential	
*ρ*	density	Kg/m^3^
σ	stress	MPa
ε	strain	%
*ζ*	phenomenological variable	
**Subscripts & acronyms**		
*0*	initial	
*e*	elastic	
MST, *μT*	Micro-Structuro-Thermal	
*p*	plastic	
*rev*	reversible	
*irr*	irreversible	
*phen*	phenomenological	
DEG	Degradation-Entropy Generation	

## References

[1] Vasudevan A.K., Sadananda K., Glinka G. (2001). Critical parameters for fatigue damage. Int. J. Fatigue.

[2] Callister W.D. (2001). Fundamentals of Materials Science and Engineering.

[3] Timoshenko S. (1940). Strength of Materials (Part I).

[4] Goodno B.J., Gere J.M. (2016). Mechanics of Materials.

[5] Hibbeler R.C. (2017). Mechanics of Materials.

[6] Shigley J.E., Mischke C.R. (1996). Standard Handbook of Machine Design.

[7] Lemaitre J., Chaboche J.-L. (1990). Mechanics of Solid Materials.

[8] Vasudevan A.K., Sadananda K., Iyyer N. (2015). Fatigue damage analysis: Issues and challenges. Int. J. Fatigue.

[9] Gomez J., Basaran C. (2005). A thermodynamics based damage mechanics constitutive model for low cycle fatigue analysis of microelectronics solder joints incorporating size effects. Int. J. Solids Struct..

[10] Gomez J., Basaran C. (2006). Damage mechanics constitutive model for Pb/Sn solder joints incorporating nonlinear kinematic hardening and rate dependent effects using a return mapping integration algorithm. Mech. Mater..

[11] Basaran C., Lin M., Ye H. (2003). A thermodynamic model for electrical current induced damage. Int. J. Solids Struct..

[12] Basaran C., Nie S. (2004). An Irreversible Thermodynamics Theory for Damage Mechanics of Solids. Int. J. Damage Mech..

[13] Basaran C., Gomez J., Gunel E., Li S., Voyiadjis G. (2014). Thermodynamic Theory for Damage Evolution in Solids. Handbook of Damage Mechanics.

[14] Amiri M., Khonsari M.M. (2010). Life prediction of metals undergoing fatigue load based on temperature evolution. Mater. Sci. Eng. A.

[15] Naderi M., Khonsari M.M. (2010). An experimental approach to low-cycle fatigue damage based on thermodynamic entropy. Int. J. Solids Struct..

[16] Naderi M., Khonsari M.M. (2010). A thermodynamic approach to fatigue damage accumulation under variable loading. Mater. Sci. Eng. A.

[17] Naderi M., Khonsari M. (2011). Real-time fatigue life monitoring based on thermodynamic entropy. Struct. Health Monit..

[18] Amiri M., Naderi M., Khonsari M.M. (2011). An Experimental Approach to Evaluate the Critical Damage. Int. J. Damage Mech..

[19] Naderi M., Khonsari M.M. (2012). A comprehensive fatigue failure criterion based on thermodynamic approach. J. Compos. Mater..

[20] Naderi M., Khonsari M.M. (2012). Thermodynamic analysis of fatigue failure in a composite laminate. Mech. Mater..

[21] Naderi M., Khonsari M.M. (2013). On the role of damage energy in the fatigue degradation characterization of a composite laminate. Compos. Part B Eng..

[22] Amiri M., Modarres M. (2014). An entropy-based damage characterization. Entropy.

[23] Naderi M., Amiri M., Khonsari M.M. (2010). On the thermodynamic entropy of fatigue fracture. Proc. R. Soc. A Math. Phys. Eng. Sci..

[24] Bryant M.D., Khonsari M.M., Ling F.F. (2008). On the thermodynamics of degradation. Proc. R. Soc. A Math. Phys. Eng. Sci..

[25] Chaboche J.L. (1989). Constitutive Equations for Cyclic Plasticity and Cyclic Viscoplasticity. Int. J. Plast..

[26] Chaboche J.L. (1991). On some modifications of kinematic hardening to improve the description of ratchetting effects. Int. J. Plast..

[27] Callen H.B. (1985). Thermodynamics and an Introduction to Thermostatistics.

[28] Doelling B.P., Ling K.L., Bryant F.F., Heilman M.D. (2000). An experimental study of the correlation between wear and entropy flow in machinery components. J. Appl. Phys..

[29] Duyi Y., Zhenlin W. (2001). A new approach to low-cycle fatigue damage based on exhaustion of static toughness and dissipation of cyclic plastic strain energy during fatigue. Int. J. Fatigue.

[30] Sosnovskiy L., Sherbakov S. (2009). Surprises of Tribo-Fatigue.

[31] Sosnovskiy L., Sherbakov S. (2016). Mechanothermodynamic Entropy and Analysis of Damage State of Complex Systems. Entropy.

[32] Osara J.A., Bryant M.D. (2019). Thermodynamics of Grease Degradation. Tribol. Int..

[33] Strutt J.W., Rayleigh B. (1877). The Theory of Sound.

[34] Onsager L. (1931). Reciprocal Relations in Irreversible processes 1. Am. Phys. Soc..

[35] Nicolis G., Prigogine I. (1977). Self-Organization in Nonequilibrium Systems.

[36] Kondepudi D., Prigogine I. (1998). Modern Thermodynamics: From Heat Engines to Dissipative Structures.

[37] Bryant M.D. (2016). On Constitutive Relations for Friction From Thermodynamics and Dynamics. J. Tribol..

[38] Bryant M.D. (2009). Entropy and Dissipative Processes of Friction and Wear. FME Trans..

[39] Osara J.A. (2017). Thermodynamics of Degradation.

[40] Osara J.A., Bryant M.D. (2019). A Thermodynamic Model for Lithium-Ion Battery Degradation: Application of the Degradation-Entropy Generation Theorem. Inventions.

[41] DeHoff R.T. (2006). Thermodynamics in Material Science.

[42] de Groot S.R. (1951). Thermodynamics of Irreversible Processes.

[43] Prigogine I. (1955). Introduction to Thermodynamics of Irreversible Processes.

[44] Bejan A. (1997). Advanced Engineering Thermodynamics.

[45] Moran M.J., Shapiro H.N. (2004). Fundamentals of Engineering Thermodynamics.

[46] Burghardt M.D., Harbach J.A. (1993). Engineering Thermodynamics.

[47] Karnopp D. (1990). Bond Graph Models for Electrochemical Energy Storage: Electrical, Chemical and Thermal Effects. J. Frankl. Inst..

[48] Bejan A. (1990). The Method of Entropy Generation Minimization. Energy and the Environment.

[49] Pal R. (2017). Demystification of the Gouy-Stodola theorem of thermodynamics for closed systems. Int. J. Mech. Eng. Educ..

[50] Glansdorff P., Prigogine I. (1971). Thermodynamic Theory of Structure, Stability and Fluctuations.

[51] Morris J.W. (2007). Notes on the Thermodynamics of Solids, Chapter 16: Elastic Solids. http://www.mse.berkeley.edu/groups/morris/MSE205/Extras/Elastic.pdf.

[52] Ramberg W., Osgood W. (1943). Description of Stress-Strain Curves by Three Parameters.

[53] Morrow J. (1965). Cyclic Plastic Strain Energy and Fatigue of Metals. Internal Friction, Damping, and Cyclic Plasticit.

[54] Kim K., Chen X., Kim K.S. (2002). Estimation methods for fatigue properties of steels under axial and torsional loading Estimation methods for fatigue properties of steels under axial and torsional loading. Int. J. Fatigue.

[55] Socie D. (1987). Multiaxial Fatigue Damage Models. ASME Trans..

[56] Budynas R.G., Nisbett J.K. (2015). Shigley’s Mechanical Engineering Design.

[57] Meneghetti G. (2007). Analysis of the fatigue strength of a stainless steel based on the energy dissipation. Int. J. Fatigue.

[58] Osara J.A. (2019). Thermodynamics of Manufacturing Processes—The Workpiece and the Machinery. Inventions.

[59] Barsom J., Rolfe S. (1999). Fracture and Fatigue Control. Structures: Applications of Fracture Mechanics.

[60] Bryant M. (2010). Unification of friction and wear. Recent Dev. Wear Prev. Frict. Lubr..

[61] Rice J.R. (1978). Thermodynamics of the quasi-static growth of Griffith cracks. J. Mech. Phys. Solids.

